# Supplementary material for: Biodegradable Block Poly(ester amine)s with Pendant Hydroxyl Groups for Biomedical Applications
Source: Polymers (Basel). 2023 Mar 16;15(6):1473. doi: 10.3390/polym15061473 (PMC10058592; doi:10.3390/polym15061473)
Supplement: Supplementary file 1 [file polymers-15-01473-s001.zip › polymers-2291422-supplementary.pdf]

polymers

Supplementary Materials

Biodegradable Block Poly(ester amine)s with Pendant Hydroxyl Groups for Biomedical Applications

Natalia Śmigiel-Gac [1], Anna Smola-Dmochowska [1], Henryk Janeczek [1] and Piotr Dobrzyński [1,2,*]

[1] Centre of Polymer and Carbon Materials, Polish Academy of Sciences, 41-819 Zabrze, Poland
[2] Faculty of Science and Technology, Jan Długosz University in Czestochowa, 13/15 Armii Krajowej Av., 42-200 Czestochowa, Poland
* Correspondence: p.dobrzynski@ujd.edu.pl

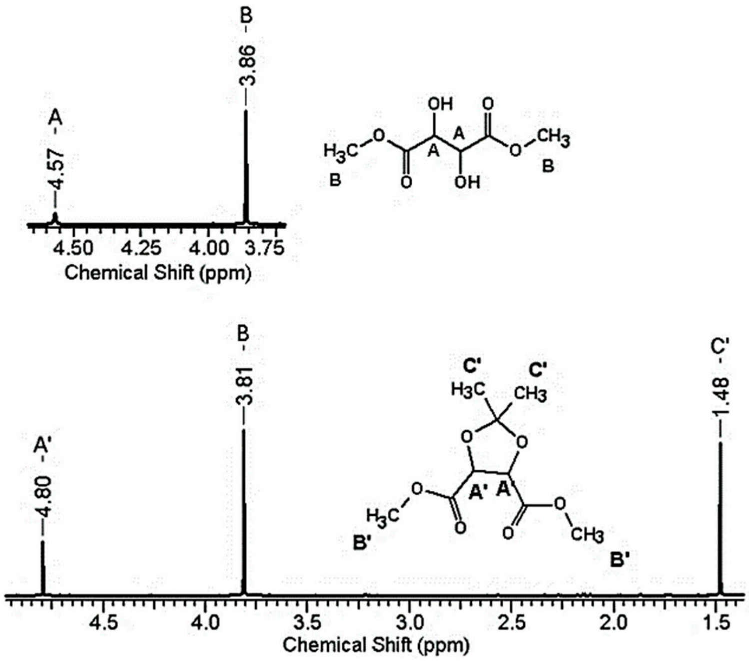

Figure S1. ^{1}H NMR spectrum of dimethyl - L tartrate and dimethyl 2,3-O-isopropylidene - L - tartrate (with protected hydroxyl groups). After protection, there are chemical shifts, signal A - C<u>H</u>OH, shift to A' -C<u>H</u>OC, B – the signal of methyl groups of the ester C<u>H</u>$_3$O, and signal C' of the -CC<u>H</u>$_3$ groups of the protecting agent.

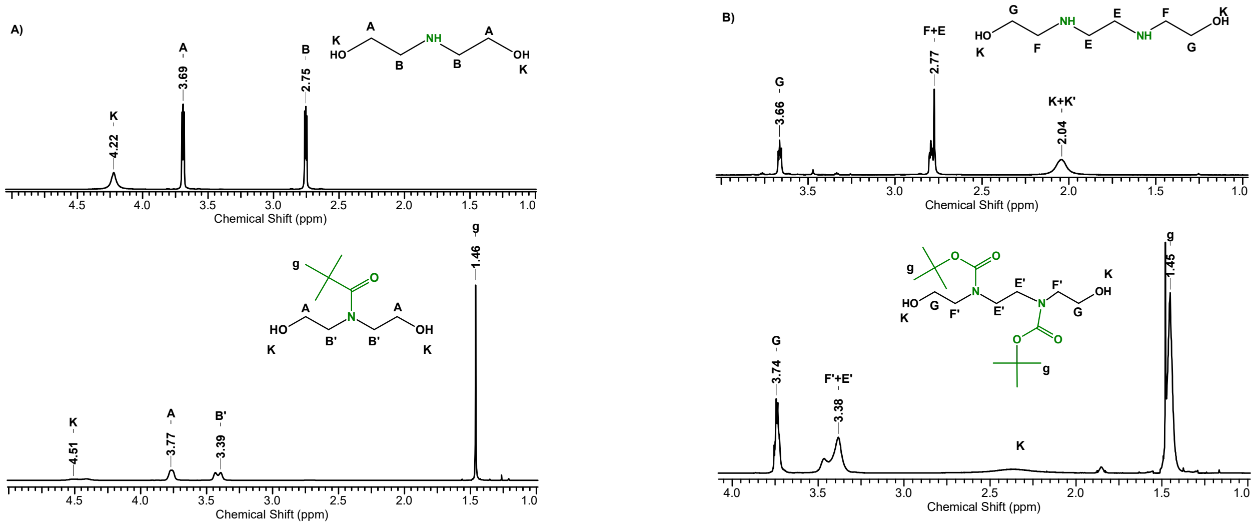

Figure S2. ^{1}H NMR spectrum, (A) diethanolamine before and after the protection of amine groups (tert-butyl bis(2-hydroxyethyl) carbamate), (B) N,N' – bis (2 hydroxyethyl) ethylenediamine before and after the protection of amino groups.

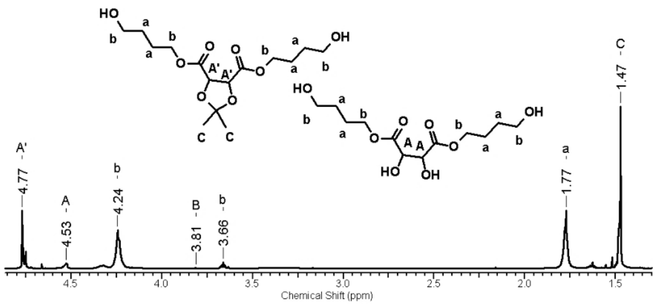

Figure S3. ^{1}H NMR spectrum of poly(butylene 2,3, - O - isopropylidene - L - tartrate) (Table 1, I1).

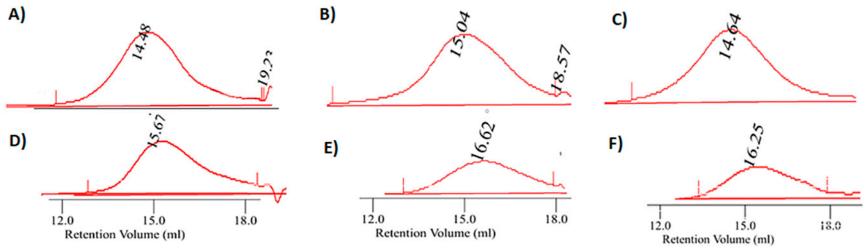

Figure S4. GPC elugrams; P6 A) before deprotection of hydroxyl and amine groups, B) after of hydroxyl and amine groups deprotection; P4 C) before deprotection of hydroxyl and amine groups D) after deprotection of hydroxyl and amine groups; P4 E) before deprotection of hydroxyl groups; F) after deprotection of hydroxyl groups.
